# P-2103. Non-invasive Group A Streptococcus Infection in the United States, 2012 – 2023: a National Reference Laboratory Retrospective Study

**DOI:** 10.1093/ofid/ofae631.2259

**Published:** 2025-01-29

**Authors:** Min Kyung Lee, Howard Engler, Rita Stainback, David Alfego, Samia Naccache

**Affiliations:** Labcorp, Durham, North Carolina; Labcorp, Durham, North Carolina; Labcorp, Durham, North Carolina; Labcorp, Durham, North Carolina; Labcorp, Durham, North Carolina

## Abstract

**Background:**

Non-invasive Group A Streptococcus (GAS) infection prevalence is not actively monitored, however understanding seasonal patterns can help inform unexpected trends in invasive and non-invasive GAS prevalence.

**Methods:**

De-identified results from 23,601,754 specimens for GAS detection from throat swabs performed at Labcorp sites from January 2012 – December 2023 were included in a retrospective, cross-sectional cohort study. 2,605,482 specimens with diagnostic tests to detect bacteria including GAS from blood culture positive bottles were included as a surrogate for invasive GAS infection (iGAS). Cross-correlation tests were used to compare the time series patterns for URT GAS and GAS bacteremia. Age-adjusted prevalence ratios comparing GAS prevalence in 2023 to 2017 were calculated using *metabin* function in the meta R package. Multivariable logistic regression models were used to test for association between patient sex, age, and geographic location and GAS infection.

**Results:**

The highest URT GAS infection levels occurred between the 8^th^ to 16^th^ weeks of the year. Higher Risk of URT GAS infection was confirmed in patients 6 – 11 years of age and 15% increased risk was observed in males. Higher positivity of non-invasive Group A Streptococcus infection was consistently observed in East North Central, Middle Atlantic and Pacific divisions of the United States. A large decrease in non-invasive Group A Streptococcus infection due to the COVID-19 pandemic was followed by a significant resurgence in late 2022 and early 2023, with return to seasonal patterns in late 2023. Prevalence of URT GAS infections in March 2023 was 55% greater than the prevalence of URT GAS infections in March 2017 (age-adjusted PR: 1.55, 95% CI: 1.52 – 1.58). iGAS positivity rates generally corresponded to URT GAS positivity rates during the same time period, including during GAS resurgence in late 2022 and early 2023.

**Conclusion:**

Data from national reference lab outpatient testing can provide potentially statistically significant infectious disease surveillance trends.

**Disclosures:**

Min Kyung Lee, PhD, Labcorp: Salary Howard Engler, PhD, D(ABMM), Labcorp: Salary|Labcorp: Stocks/Bonds (Public Company) Rita Stainback, MS, MT (ASCP) SM, DLM, Labcorp: Salary David Alfego, PhD, Labcorp: Employee|Labcorp: Stocks/Bonds (Public Company) Samia Naccache, PhD, D(ABMM), Labcorp: Salary|Labcorp: Stocks/Bonds (Public Company)

